# A UNet++-Based Approach for Delamination Imaging in CFRP Laminates Using Full Wavefield

**DOI:** 10.3390/s25144292

**Published:** 2025-07-09

**Authors:** Yitian Yan, Kang Yang, Yaxun Gou, Zhifeng Tang, Fuzai Lv, Zhoumo Zeng, Jian Li, Yang Liu

**Affiliations:** 1 State Key Laboratory of Precision Measuring Technology and Instruments, Tianjin University, Tianjin 300072, China; 2019205372@tju.edu.cn (Y.Y.); gouyaxun@163.com (Y.G.); zhmzeng@tju.edu.cn (Z.Z.); 2 Department of Electrical and Computer Engineering, University of Florida, Gainesville, FL 32611, USA; yang.kang@ufl.edu; 3 Institute of Modern Manufacturing Engineering, Zhejiang University, Hangzhou 310058, China; tangzhifeng@zju.edu.cn (Z.T.); lfzlfz@zju.edu.cn (F.L.)

**Keywords:** ultrasonic guided wave, non-destructive evaluation, carbon fiber-reinforced plastic, deep learning, delamination

## Abstract

The timely detection of delamination is essential for preventing catastrophic failures and extending the service life of carbon fiber-reinforced polymers (CFRP). Full wavefields in CFRP encapsulate extensive information on the interaction between guided waves and structural damage, making them a widely utilized tool for damage mapping. However, due to the multimodal and dispersive nature of guided waves, interpreting full wavefields remains a significant challenge. This study proposes an end-to-end delamination imaging approach based on UNet++ using 2D frequency domain spectra (FDS) derived from full wavefield data. The proposed method is validated through a self-constructed simulation dataset, experimental data collected using Scanning Laser Doppler Vibrometry, and a publicly available dataset created by Kudela and Ijjeh. The results on the simulated data show that UNet++, trained with multi-frequency FDS, can accurately predict the location, shape, and size of delamination while effectively handling frequency offsets and noise interference in the input FDS. Experimental results further indicate that the model, trained exclusively on simulated data, can be directly applied to real-world scenarios, delivering artifact-free delamination imaging.

## 1. Introduction

Carbon fiber-reinforced polymers (CFRP) are extensively used in modern engineering applications due to their advantages, which include low weight, high strength, and design versatility [1,2,3]. However, various factors such as external stress, manufacturing defects, etc., may lead to damage in the form of fiber fracture, matrix cracking, delamination, etc. [4,5,6]. To prevent catastrophic failures and prolong the service life of CFRP materials, the early detection of such damage is crucial. Common non-destructive evaluation (NDE) methods include visual inspection, ultrasonic testing, acoustic emission, electromagnetic techniques, thermal imaging, and optical testing [7,8,9]. Among these, guided waves have shown great promise for damage detection in thin-walled structures due to their long propagation range and high sensitivity [10]. However, the complexity of guided waves—stemming from their multimodal nature and dispersive characteristics—makes the interpretation of guided wave signals highly challenging. Traditional guided wave NDE techniques often rely on the operator’s expertise and require substantial domain knowledge to accurately interpret the results.

The rapid advancement of deep learning (DL) technology has brought about innovative solutions in the field of NDE. By replacing the manual feature extraction and analysis typical of traditional methods with a data-driven, end-to-end prediction framework, DL models can autonomously identify underlying patterns in the ultrasonic data and predict damage conditions.

Recent research has shown that DL techniques have demonstrated significant promise in detecting defects in laminated composite materials [11,12]. Zhang et al. [13] employed the finite element method (FEM) to simulate the detection of 126-layer CFRP with wrinkles using an ultrasonic phased array probe. They transformed the acquired time-domain signals into 2D images via the short-time Fourier transform and trained a convolutional neural network (CNN) to classify various wrinkle conditions. Cheng et al. [14] developed an A-scan signal dataset and trained CNN, long short-term memory (LSTM), and CNN-LSTM models to estimate the depth of impact damage, achieving a minimum error of 8%. Feng et al. [15] used the time of flight of scattered waves generated by simulated delamination with magnetic blocks as input to a fully connected network for damage localization, with an error as low as 4.6 mm. Su et al. [16] employed a CNN to both detect delamination and classify impact energy. They trained the model using frequency domain features extracted through Fourier transform. Their results indicated that the CNN could successfully classify the impact energy level and predict the delamination position, correctly classifying and localizing 191 out of 192 samples. Rautela et al. [17] used continuous wavelet transform to create 2D time–frequency domain images for CNN training, successfully predicting the lateral position and length of delamination with average relative errors of 8.5% and 11.25%, respectively. Yang et al. [18] trained a CNN using fuzzy images derived from algebraic reconstruction technique and conducted tests on delamination damages, significantly reducing image artifacts. Zhang et al. [19] introduced a convolutional sparse coding UNet (CSCUNet) to enhance the resolution of blurry images produced by delay and sum (DAS) methods. They collected guided wave signals from 28 sensing paths within a sensor array of eight piezoelectric sensors surrounding the damage. These signals were processed using DAS and binarization to generate preliminary images for model training. Their CSCUNet model outperformed traditional UNet approaches in delamination imaging. Ijjeh et al. [20,21] pioneered the application of the root mean square (RMS) spectrum of the full wavefield for model training, employing an end-to-end pixel image segmentation approach to identify delamination’s position, size, and shape. Their models, trained on purely simulated data, demonstrated remarkable accuracy in imaging delamination in experimental scenarios, showcasing excellent generalization ability.

The aforementioned studies highlight the significant potential of DL-based NDE techniques for application in CFRP materials. However, several critical challenges persist. First, while damage detection and localization have been widely explored, damage imaging—a rapidly evolving research area—still faces challenges in terms of accuracy. Second, creating diverse and representative datasets is costly. The data collection process is limited by experimental setups, and the high time, cost, and manpower required for sample fabrication and extensive testing further exacerbate this issue. Third, the generalization capability of DL models remains a key hurdle. Although numerical simulation techniques are often employed to reduce dataset creation costs, models trained exclusively on simulation data still encounter difficulties when applied directly to real-world measurements.

To tackle the challenges outlined above, this paper presents a delamination imaging method based on UNet++ for CFRP. A dataset was created using FEM, capturing full wavefields from the surfaces of 16-layer CFRP with delamination damage of varying positions, sizes, and shapes. The frequency domain energy distribution was extracted as input for the model, and UNet++ was utilized to perform end-to-end mapping from the frequency domain spectrum (FDS) to the delamination image. The model’s performance was evaluated using a self-constructed simulation dataset, a sample obtained from a scanning laser Doppler vibrometry (SLDV) experiment, and an open dataset developed by Kudela and Ijjeh [22].

The contributions and innovations of this work in the quantitative NDE of CFRP can be summarized in the following three key aspects:

(1) UNet++ is employed to segment the energy distribution in 2D FDS, achieving end-to-end artifact free delamination imaging.
(2) The model trained with multi-frequency FDS demonstrates accurate delamination imaging, even in scenarios with input frequency offsets, while also exhibiting good noise resistance.
(3) The model trained on pure simulation dataset can be used to predict experimental samples with high accuracy. It provides a solution for quantitative detection of invisible delamination damage in CFRP.

## 2. Dataset Preparation

### 2.1. Material Properties of Specimen

**Figure 1:**
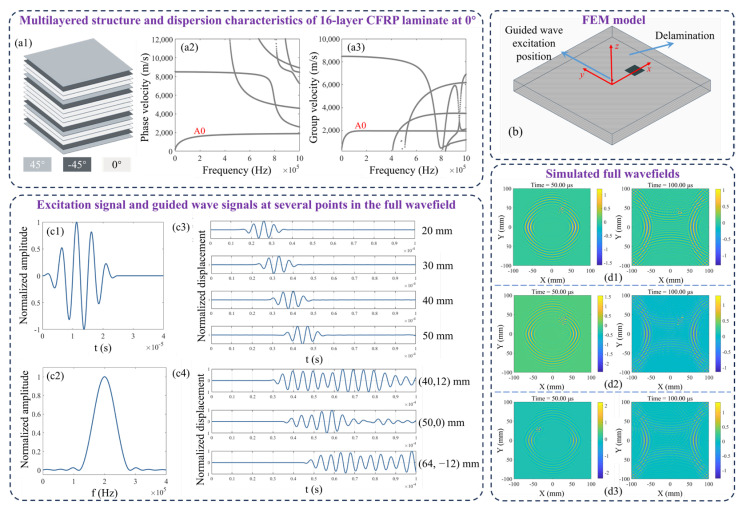
Dispersion characteristics and numerical simulation of the full wavefields. (**a1**) shows the laminated structure of the CFRP, with the SAFE method applied to compute its (**a2**) phase velocity and (**a3**) group velocity dispersion curves. (**b**) shows a schematic diagram of the FEM model. The 200 kHz excitation signal applied at the center of the upper surface is shown in (**c1**), and its frequency domain components are displayed in (**c2**). (**c3**) illustrates the out-of-plane displacement at several points along the *x*-axis of the damage-free plate surface, which aligns with the A0 mode. (**c4**) illustrates the out-of-plane displacement at the center of a 20 mm side length square delamination located at three different positions, demonstrating that the GW are trapped within the delamination regions, leading to localized energy accumulation. Snapshots of the wavefields influenced by (**d1**) circular, (**d2**) square, and (**d3**) elliptical delamination are provided.

The specimen is a 16-layer CFRP laminate with fiber orientations of [+45/−45/0/0]_2s_, and a total thickness of 2 mm, as shown in Figure 1(a1). The material properties of a single layer are as shown in Table 1.

The dispersion curves of the 16-layer CFRP laminate were calculated using the semi-analytical finite element (SAFE) method [23], with the results in the 0° direction presented in Figure 1(a2,a3).

### 2.2. Numerical Simulation

Based on the laminate structure and material properties, a 3D FEM model centered at the origin of the Cartesian coordinate system was established without applying absorbing boundary conditions, which allowed boundary reflections to be retained. The model dimensions are 200 × 200 × 2 mm^3^ in length, width, and thickness, as shown in Figure 1b. Delamination damage occurs between the fourth and fifth layers. To provide a diverse range of samples, circular, square, and elliptical damage shapes were defined, with each shape’s dimensions (side length, diameter, and major/minor axis) varying within the range of 16 mm to 24 mm. Additionally, the defect locations were randomly varied within the x-y plane.

The excitation signal is applied at the center of the upper surface:

(1) $$v(t)=v_{pp}\left[H(t)-H(t-\frac{N}{f_{c}})\right]\times (1-\cos\frac{2\pi f_{c}t}{N})\sin2\pi f_{c}t$$

where *v_pp_* represents peak-to-peak amplitude of the signal, *H*(*t*) is the Heaviside step function, *f_c_* is the excitation frequency, and *N* is the number of cycles.

As evident from the dispersion curves, when the excitation frequency is below 400 kHz, only two modes, A0 and S0, are present. Considering the sensitivity of guided waves to damage and wave attenuation, a 200 kHz guided wave was excited at the center of the upper surface. Figure 1(c1,c2), respectively, depict the excited signal in the time domain and frequency domain.

Figure 1(c3) illustrates the out-of-plane displacement of uniformly distributed points along the *x*-axis on the surface of an undamaged plate, which aligns with the A0 mode. Figure 1(c4) illustrates the out-of-plane displacement at the center of a 20 mm side length square delamination located at three different positions. It can be observed that the guided waves are trapped within the delamination regions, leading to localized energy accumulation. A total of 450 distinct full wavefields were generated, uniformly encompassing three types of delamination shapes. Each wavefield consists of time series signals spanning 200 time steps, recorded at 512 × 512 uniformly distributed coordinate points over a 200 × 200 mm^2^ area. Representative snapshots of wavefields influenced by circular, square, and elliptical delamination are depicted in Figure 1(d1), (d2), and (d3), respectively.

### 2.3. Frequency Domain Spectra Extraction

In this study, a 2D fast Fourier transform (FFT) [24] was used to extract the energy distribution:

(2) $$F(u,v,f)=\int_{-\infty }^{\infty }\int_{-\infty }^{\infty }f(x,y,t)\;e^{-j2\pi (ux+vy)}dxdy$$

where *f*(*x*, *y*, *t*) is the time series signal in the 2D spatial domain. *F*(*u*, *v*, *f*) is the 2D FDS. *u* and *v* are the two frequency variables, corresponding to the horizontal and vertical directions, and *j* is the imaginary unit.

**Figure 2:**
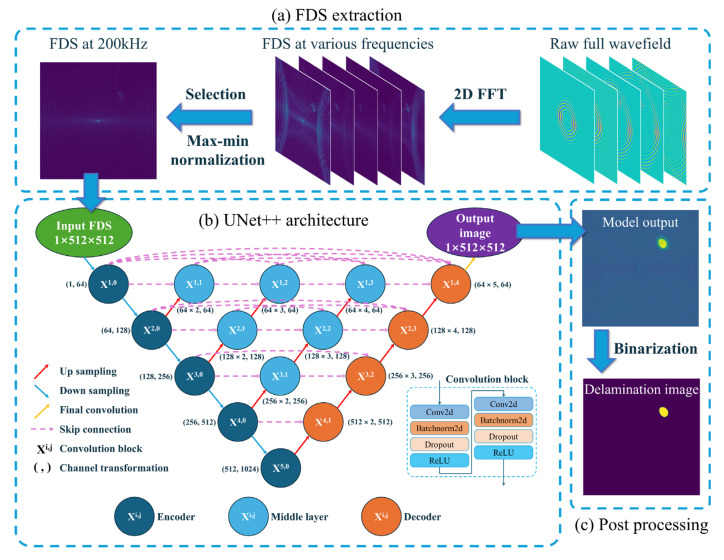
Delamination damage imaging method utilizing UNet++ and 2D FDS, including (**a**) FDS extraction, (**b**) UNet++ architecture, and (**c**) post-processing.

This approach requires the guided wave to propagate across the entire test area. After applying a 2D FFT, the full wavefield data captured from the structure’s surface is transformed into 2D FDS at various frequencies. As the energy of the excited wave is predominantly concentrated around 200 kHz, the FDS at this frequency was selected and each FDS was individually normalized using min–max scaling. Consequently, the original 3D full wavefield data (201 × 512 × 512) was compressed into a 2D 200 kHz FDS (512 × 512) for model training. The above process is shown in Figure 2a. Compared to training models directly on raw full wavefield data, employing FDS significantly reduces the number of computational parameters in the neural network, thereby decreasing computational costs and improving training efficiency.

### 2.4. Flipping Augmentation

Flipping augmentation is commonly used in various DL-based visual tasks to efficiently expand dataset size [25]. In this study, horizontal, vertical, and diagonal flipping were applied to the 200 kHz FDS. This technique rapidly generated simulated delamination scenarios at three new locations by spatially transforming existing samples, thereby increasing the diversity of delamination positions and shapes within the dataset [20,21]. As a result, the size of the simulation dataset was quadrupled, ultimately containing 1800 samples.

### 2.5. Dataset Labeling and Splitting

Binary images were created as ground truth labels. The pixel values in these images were derived from the projection of the delamination onto the x-y plane. These projections were discretized into 512 × 512 uniformly spaced pixels, where a value of 0 indicates no damage at the pixel location, and a value of 1 indicates the presence of damage.

The augmented dataset was split into three parts: the training set accounts for 70%, the validation set accounts for 20% and is used for generalization evaluation during the training process, and the test set accounts for 10% and is used for final performance evaluation. The outcome of the dataset splitting is summarized in Table 2.

## 3. Delamination Imaging Model Construction, Training, and Evaluation Methods

### 3.1. Neural Network

In this study, UNet++ [26] is employed to process FDS data, leveraging its high-performance image segmentation capabilities. UNet++ is an enhanced version of the widely used UNet architecture [27], originally designed for medical image segmentation. The architecture of UNet++ is illustrated in Figure 2b. In this model, X^i,j^ denotes a convolution module consisting of two sequential layers: “convolution-batch normalization-dropout-ReLU”. Each convolutional layer uses a kernel size of 3 × 3, a stride of 1, and a padding of 1, with a dropout rate of 0.2. Down sampling is performed using a max pooling layer with a kernel size of 2 × 2 and stride of 2. Up sampling is achieved through transpose convolution, also with a kernel size of 2 × 2 and stride of 2. At the model’s output, a final convolutional layer reduces the channel depth to 1, with a kernel size of 1 × 1 and stride of 1. The encoder’s features are passed through multiple progressively refined sub-networks before being forwarded to the decoder, which helps to retain more semantic information and effectively process multi-scale features [26].

### 3.2. Training Strategy

The Adam optimizer [28], which integrates the benefits of Momentum and RMSProp, along with the Cosine Annealing Learning Rate (CALR) scheduler [29], were employed to dynamically adjust the learning rate (LR) during model training, thereby accelerating convergence, with an initial LR of 0.001. The Adam optimizer automatically adapts the LR by computing individual adaptive learning rates for each parameter, based on both first-order moment estimates (the mean) and second-order moment estimates (the uncentered variance). Meanwhile, CALR uses a cosine annealing strategy to gradually reduce the LR towards zero, aiding in the fine-tuning of the model during the later stages of training.

### 3.3. Performance Evaluation Methods

This section defines two evaluation metrics for the model: a loss function used to control model convergence, and an accuracy metric used to assess the quality of the imaging results.

To effectively evaluate imaging performance during model training, we integrated two widely used loss functions in DL: Binary Cross-Entropy Loss (BCE Loss) [30] and Dice Loss [31]. This combination addresses both pixel-level classification errors and the overlap between predicted and ground truth images. The resulting loss function is termed “BCE Dice Loss” (BCED).

The BCE Loss integrates the sigmoid activation function [32], primarily focusing on the classification accuracy of each individual pixel. The sigmoid function transforms the model’s raw outputs into probabilities, ensuring that the results fall within the 0 to 1 range. The calculation of BCE Loss is performed as follows:

(3) $$BCE\;Loss=-\frac{1}{N}\sum_{i=1}^{N}\left[t_{i}\log(\sigma (p_{i}))+(1-t_{i})\log(1-\sigma (p_{i}))\right]$$

where *t_i_* is the ground truth of i-pixel, *p_i_* is predicted value of i-pixel, and *N* is the total number of pixels.

Dice Loss is based on the Dice coefficient and is used to measure the overlap between predicted and true labels. The formula for the Dice coefficient is as follows:

(4) $$Dice\;Coefficient=\frac{2\sum_{i=1}^{N}p_{i}\;t_{i}\;+smooth}{\sum_{i=1}^{N}p_{i}\;+\sum_{i=1}^{N}t_{i}+smooth}$$

where *smooth* is a constant, usually set to 1, to avoid situations where the denominator is zero. Dice Loss is a complementary form of the Dice coefficient:

(5) Dice Loss=1−Dice Coefficient

The BCED combines the contributions of BCE Loss and Dice Loss by summing them:

(6) BCED=BCE Loss+Dice Loss

By minimizing BCED, the model’s predictions are adjusted to align more closely with the ground truths.

The Intersection over Union (IoU) was used to assess the accuracy of the imaging results. It is a widely used metric for evaluating the consistency between the model’s predicted results and the ground truth labels in image segmentation or object detection tasks. The IoU is defined as follows:

(7) $$IoU=\frac{p_{i}^{\tau }\cap t_{i}}{p_{i}^{\tau }\cup t_{i}}$$

where $p_{i}^{\tau }$ is the binarized prediction of the model, which is calculated by the following:

(8) $$p_{i}^{\tau }=\left\{\begin{array}{c} 1\;\;\;\;\;\;\;\;\;\;\;\;\;\;\;\;p_{i}\geq \tau \\ 0\;\;\;\;\;\;\;\;\;\;\;\;\;\;\;\;p_{i}<\tau \end{array}\right.$$

where *τ* is threshold, and is set to 0.5, which is the midpoint of the BCEL probability. Figure 2c illustrates an example of binarizing the model output.

Based on its definition, IoU has the following two key characteristics:

(1) Penalty for displacement: If there is a spatial offset in the predicted area, the IoU will decrease significantly, even if the size of the predicted area is correct.
(2) Penalty for redundancy: If the prediction includes areas that do not correspond to the target (i.e., non-target areas), the IoU will decrease significantly, even if the target area is correctly covered.

Therefore, IoU provides a comprehensive evaluation of delamination imaging results, taking into account the location, size, and shape of the predicted damage area.

## 4. Results and Discussion

**Figure 3:**
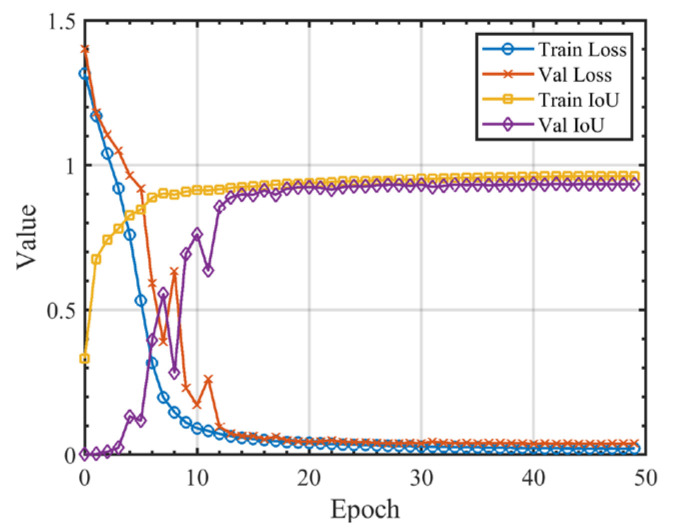
Convergence process of UNet++, illustrating the changes in BCED and mean IoU for the training and validation sets over 50 epochs.

The model training was conducted on an NVIDIA RTX 4090 GPU for 50 epochs, with the convergence process illustrated in Figure 3. The loss and mean IoU curves for both the training and validation sets stabilize around the 40th epoch. During the subsequent 10 epochs, the validation loss shows no change greater than 0.001, indicating that the model had fully converged by the 50th epoch.

### 4.1. Generalization in the Simulation Test Set

The UNet++ model was first tested on 180 unseen samples from the simulation test set and compared with the results of the traditional Wavenumber Filtering (WF) method [33]. In the WF method, the full wavefield data undergoes 3D FFT, filtering, and inverse FFT. The filtered FDS at 200 kHz is then selected and binarized. A circular area with a center diameter of 24 pixels is removed from the wavefield to eliminate the influence of the ultrasound source. In both methods, a threshold of 0.5 is applied to ensure a fair comparison. In addition, we marked the IoU score of the delamination images in the lower right corner.

**Figure 4:**
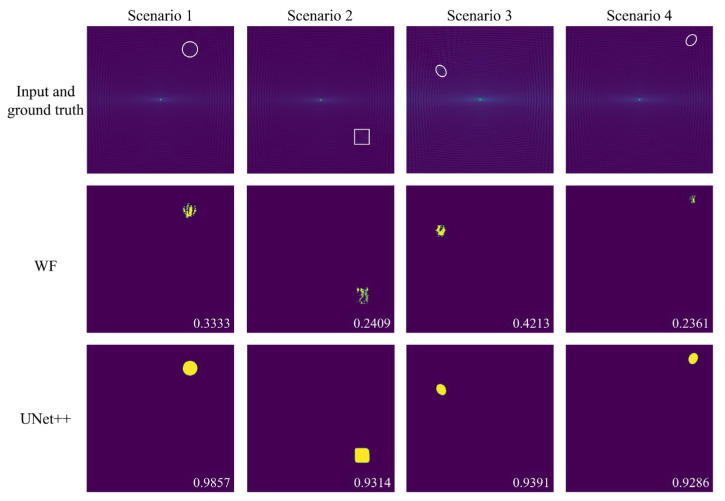
Delamination imaging results of WF and UNet++ in four exemplary scenarios. The first row shows the input of the model and the actual delamination position, shape, and size (the region marked by white line), while the second and third row, respectively, display the delamination imaging results of WF and UNet++.

Figure 4 presents the delamination imaging results for both WF and UNet++ across four exemplary scenarios. The results clearly show that UNet++ effectively maps FDS to delamination images, surpassing the performance of WF. This conclusion is further corroborated by the statistical IoU values in Table 3, which summarize the mean, maximum, minimum, and standard deviation (SD) of the IoU scores for the delamination imaging results of both WF and UNet++ in the entire test set. The data demonstrates that UNet++ outperforms WF across all statistical metrics, highlighting its superior imaging accuracy and stability. The average processing time for a single sample using UNet++for delamination imaging is 0.0797s.

It is worth mentioning that among the four exemplary scenarios, the IoU of delamination imaging in scenario 4 achieved by UNet++ is the lowest. This can be attributed to the proximity of the delamination to the structure’s corner, where the frequency domain energy components from edgewave reflections interfere with UNet++’s segmentation performance.

### 4.2. Generalization on Frequency Offset FDS

This section assesses the generalization ability of UNet++ when the input FDS experiences a frequency offset, also serving as an evaluation of its adaptability in application.

Because the guided wave excitation signal has a bandwidth covering approximately 120 kHz to 280 kHz, as illustrated in Figure 1(c2), we could extract spectral energy at discrete frequency slices from *F*(*u*, *v*, *f*) to generate new FDS samples. FDS at frequencies of 140 kHz, 160 kHz, 180 kHz, 220 kHz, 240 kHz, and 260 kHz were extracted from the full wavefield cases in the test set, generating six new test sets (180 cases each). These frequency offset FDS were then fed into the trained UNet++ for evaluation.

Additionally, we randomly selected FDS at specific frequencies from each full wavefield case in the training set, compiled them into a remixed training set, and retrained UNet++ using this set for comparison. The remixed training set introduces spectral variations due to frequency offsets, with the goal of enhancing the model’s generalization ability on frequency offset FDS. Table 4 presents the composition of the remixed training set. For each case in the training set, one frequency was randomly selected from seven candidates (140 kHz, 160 kHz, 180 kHz, 200 kHz, 220 kHz, 240 kHz, and 260 kHz), which results in slight differences in the final number of FDS samples per frequency.

**Figure 5:**
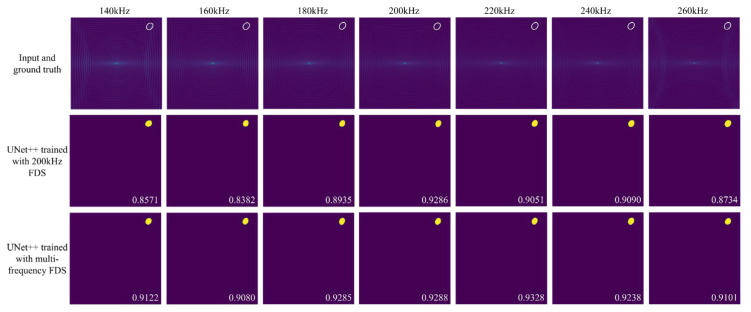
Delamination imaging results for various frequency FDS in scenario 4, comparing UNet++ trained with 200 kHz FDS and UNet++ trained with multi-frequency FDS. The first row displays the model input and ground truth, the second row shows the delamination images predicted by UNet++ trained with 200 kHz FDS, and the third row presents the delamination images predicted by UNet++ trained with multi-frequency FDS.

**Figure 6:**
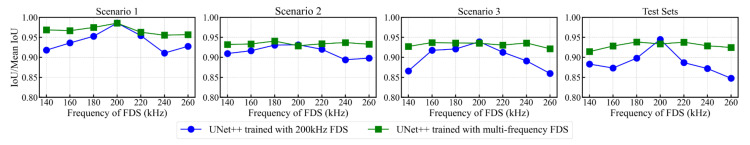
Variation in IoU of delamination imaging results for various frequency FDS across scenario 1, scenario 2, and scenario 3, along with the mean IoU for various test sets composed of specific frequency FDS, comparing UNet++ trained with 200 kHz FDS and UNet++ trained with multi-frequency FDS.

Figure 5 presents the delamination imaging results for frequency offset FDS in scenario 4, comparing UNet++ trained with 200 kHz FDS and UNet++ trained with multi-frequency FDS. The results show that UNet++ trained solely on 200 kHz FDS can directly process frequency offset FDS to achieve accurate delamination imaging. However, as the frequency shifts further, the imaging accuracy declines significantly. In contrast, after retraining with multi-frequency FDS, the accuracy of UNet++ in predicting delamination images from frequency offset FDS improves significantly, with the enhancement becoming more pronounced as the frequency offset increases. This trend is further corroborated by the results in Figure 6, which illustrates the IoU of delamination imaging for frequency offset FDS across scenarios 1, 2, and 3, along with the mean IoU for various test sets composed of specific frequency offset FDS. It is evident that UNet++ trained solely on 200 kHz FDS is more specialized for the 200 kHz scenario, giving it a slight advantage in processing 200 kHz FDS. However, the model trained with multi-frequency FDS achieves significantly higher overall accuracy across various frequency scenarios. These results are consistent with our expectations, as the model retrained with multi-frequency FDS has learned to adapt to variations in FDS caused by frequency offsets, enabling it to perform better in such scenarios. It can be observed that for a given scenario, models trained with multi-frequency FDS achieve similar IoU scores at different frequencies, while the frequency that produces the highest IoU varies. This is likely due to the balanced distribution of FDS samples across frequencies in the training dataset, which results in the model not focusing primarily on features of any specific frequency, and thus not achieving peak performance at a specific frequency.

### 4.3. Robustness Under Noise Interference

Due to the idealized conditions of the simulation, the purely simulated wavefields do not accurately reflect actual measurements, where factors such as sensor noise and environmental noise inevitably introduce disturbances [34]. This section evaluates test data consisting of simulated wavefields with various types of added noise, which serves to mimic the noise interference present in real-world measurements.

Three different types of noise—white Gaussian noise, white Laplace noise, and pink noise—were synthesized, and the signal-to-noise ratio (SNR) is used to represent the intensity of the noise. Based on the commonly observed SNR range of 20 dB to 50 dB in practical guided wave measurements [17], noise levels of 20 dB, 30 dB, and 40 dB were added to the full wavefield cases in the test set. These noisy full wavefields were then converted into 2D FDS and fed into the trained UNet++, which was trained with multi-frequency FDS (noiseless data), for testing.

**Figure 7:**
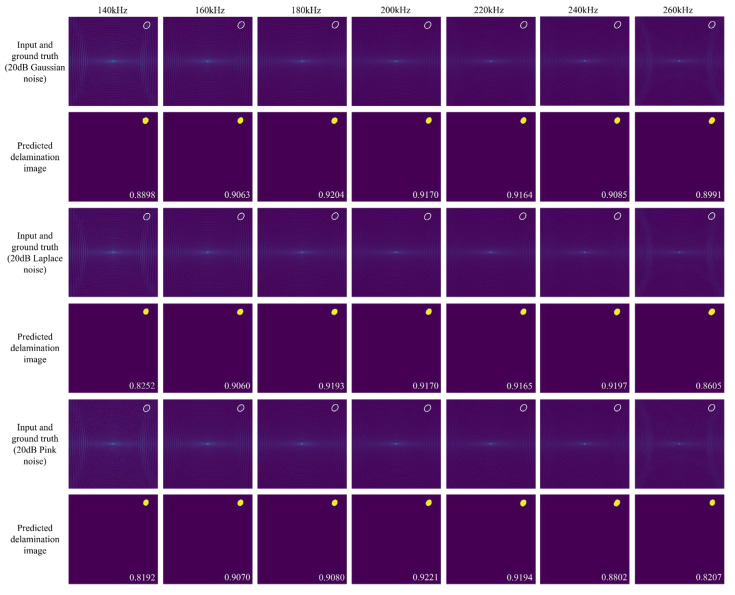
Delamination imaging results for various types of 20 dB noise-interfered FDS in scenario 4, using UNet++ trained with multi-frequency FDS. The first, third, and fifth rows display the model inputs and ground truth, including FDS with various types of 20 dB noise interferences. The second, fourth, and sixth rows show the predicted delamination images.

**Figure 8:**
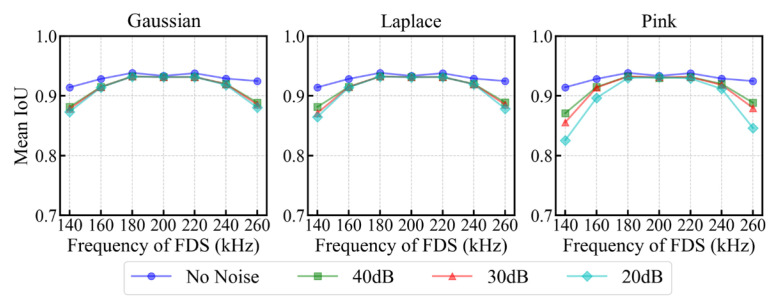
Variation in mean IoU for various test sets composed of specific frequency FDS with varying types and levels of noise interference.

Figure 7 presents the delamination imaging results for various types of 20 dB noise-interfered FDS in scenario 4. The results demonstrate that the model effectively resisted interference from different types of synthetic noise, maintaining accurate delamination imaging without introducing artifacts. This robustness can be attributed to the overall trend of FDS being maintained well after adding noise, as well as the inherent robustness of UNet++. The decrease in IoU due to noise interference is mainly observed in the predicted delamination images of FDS with large frequency offsets (140 kHz and 260 kHz). This trend is further supported by the results shown in Figure 8, which illustrates the variation in mean IoU for different test sets composed of specific frequency FDS that have been interfered with 20 dB, 30 dB, and 40 dB white Gaussian noise, white Laplace noise, and pink noise. The impact of noise interference is less pronounced in 200 kHz FDS and those with small frequency offsets (180, 220 kHz), as the guided wave energy at these frequencies is relatively strong (as shown in Figure 1(c1)), making them less susceptible to noise. In contrast, the IoU of delamination images for the FDS with further frequency offsets decreases more significantly. This is attributed to the weaker energy of the guided waves at these offset frequencies, where noise becomes more prominent, introducing additional bright spots that interfere with the model’s ability to segment the energy changes caused by delamination.

### 4.4. Experimental Test

In this section, the data obtained from the SLDV experiment is used to evaluate the generalization capability of the delamination imaging model in real-world measurements.

**Figure 9:**
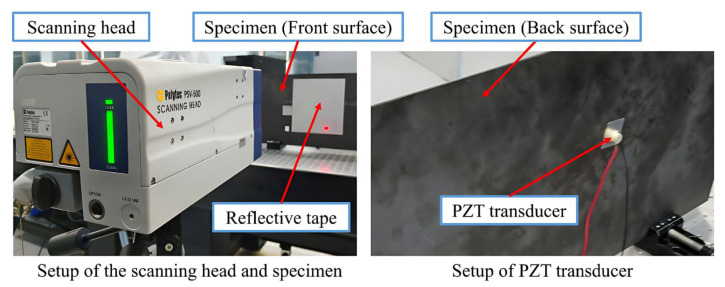
Experimental setup.

Figure 9 shows the experimental setup. The geometric dimensions of the experimental CFRP (T300) plate are 700 × 300 × 2 mm^3^, and the lay-up configuration and material properties are consistent with the numerical simulation. During the manufacturing process between the fourth and fifth layers, a 20 × 20 mm^2^ square polytetrafluoroethylene insert with thickness of 0.01 mm was introduced to simulate delamination. Guided waves were excited by a PZT transducer installed on the back of the plate. The excitation signal was a 200 kHz sine wave with five cycles (90 Vpp), and a sampling interval of 10 ms was set to ensure that there was no interference between each excitation signal. The scanning step was 1mm and the sampling rate was 7.8125 MHz. The FDS calculated from experimental data were further interpolated to 512 × 512 to fit model input.

**Figure 10:**
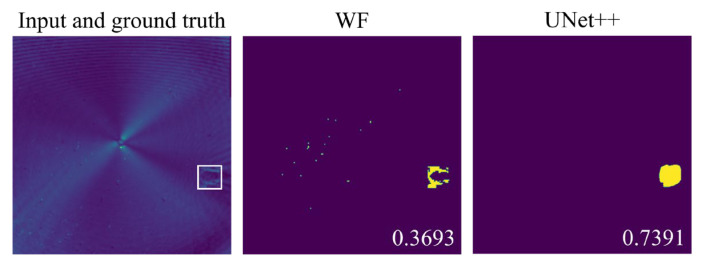
200 kHz FDS calculated from the full wavefield obtained through SLDV measurements, along with delamination imaging results using WF and U-Net++.

The experimental sample was fed into the UNet++ model trained with purely simulation data (multi-frequency FDS). Figure 10 presents the delamination imaging results for both WF and UNet++ in the 200 kHz scenario. The 200 kHz FDS reveals high-brightness noise points, which are attributed to factors like defects in the adhesive bonding of reflective paper and electrical noise, and these are clearly visible in the WF imaging results. In contrast, UNet++ effectively mitigates the interference from these noise points, achieving artifact-free imaging with an IoU of 0.7391.

**Figure 11:**
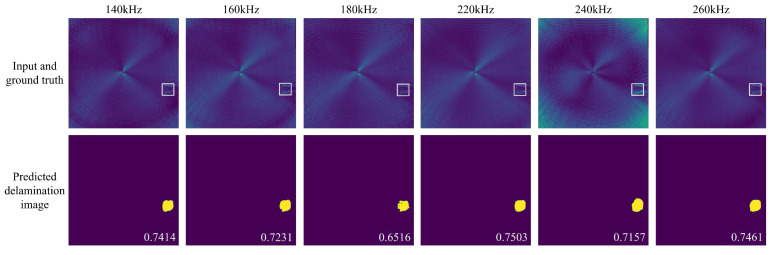
Delamination imaging results for frequency offset FDS in the experimental scenario, using UNet++ trained with simulation multi-frequency FDS. The first row displays the model input and ground truth; the second row shows the predicted delamination images.

Figure 11 displays the results of UNet++ handling frequency offset FDS. It is evident that UNet++ demonstrated strong generalization on frequency offset FDS in the experimental scenario, achieving a maximum IoU of 0.7503. Given that the model was trained exclusively on pure simulation data, these experimental results further validate the practical potential of the proposed delamination imaging method.

### 4.5. Generalization on Another Dataset

In this section, we evaluate the delamination imaging model using the open dataset built by Kudela and Ijjeh [22]. Additionally, we compare our model with the methods reported by Ijjeh et al. [20,21], where multiple DL models were trained using RMS. To ensure a fair comparison, the same dataset was used under the following standardized conditions: (1) The dataset was augmented through horizontal, vertical, and diagonal flips, increasing the dataset size by a factor of four. (2) The augmented dataset was randomly shuffled and split into 80% for training and 20% for testing. (3) Five-fold cross-validation was applied to the training set, and the mean IoU score across all folds was calculated for comparison.

The open dataset contains 475 simulated full wavefield cases of Lamb wave propagation in CFRP plates, each with dimensions of 500 × 500 × 3.9 mm^3^. The delaminations are positioned between the third and fourth layers of the CFRP structure, varying in terms of position, shape, and size. Guided waves are generated at the center of the plate using an equivalent piezoelectric force applied as a sinusoidal signal modulated by a Hann window, with a carrier frequency of 50 kHz.

In the studies by Ijjeh et al. [20,21], full wavefield data with a duration of 750 μs were used to calculate the RMS spectra, where the guided waves reflected off the boundary and returned twice to the center of the plate. In contrast, this paper utilized wavefield data with a shorter duration of 468.75 μs to extract the FDS, capturing the guided wave propagation over the entire plate with just a single pass. This approach reduces the computational cost associated with dataset construction by minimizing simulation parameters.

**Figure 12:**
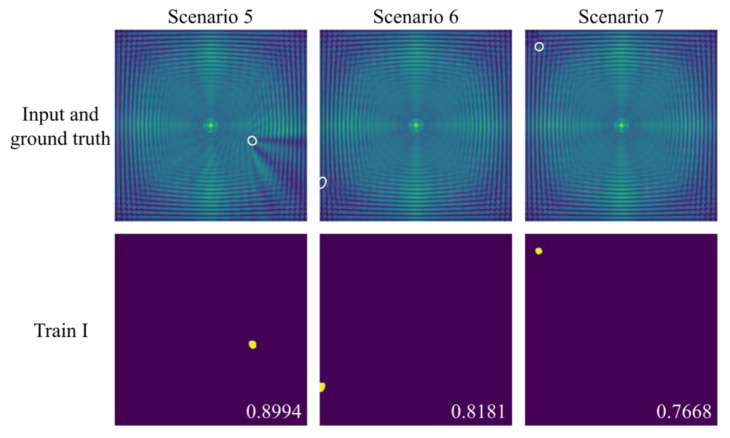
Delamination imaging results in scenario 5 to 7. The first row shows the input of the model and the ground truth, while the second row displays the delamination imaging results of UNet++.

UNet++ was initially trained using 50 kHz FDS (Train I). Figure 12 presents the delamination imaging results of the model in Train I across three representative scenarios. In scenarios 6 and 7, where the delamination is located near the edge or corner, the IoU is lower. This reduction can be attributed to the interference caused by the frequency domain energy components of edge wave reflections, which affect UNet++’s segmentation performance. This trend aligns with the findings from scenario 4 and the results reported by Ijjeh et al. [20,21]. Notably, in scenario 7, where the delamination is situated in the upper-left corner of the plate, the wave reflections from both the left and upper edges contribute to the interference, resulting in the lowest IoU among the three scenarios.

Subsequently, we assessed the generalization capability of the model on frequency offset FDS. FDS were extracted from the full wavefield cases in the test set at frequencies of 40 kHz, 45 kHz, 55 kHz, and 60 kHz, resulting in four distinct test sets, each comprising 380 cases. Furthermore, FDS at these specific frequencies were randomly selected from the full wavefield cases in the training set to construct a remixed training set, as summarized in Table 5. This remixed training set was then used to retrain UNet++ (Train II), with 5-fold cross-validation applied as well.

**Figure 13:**
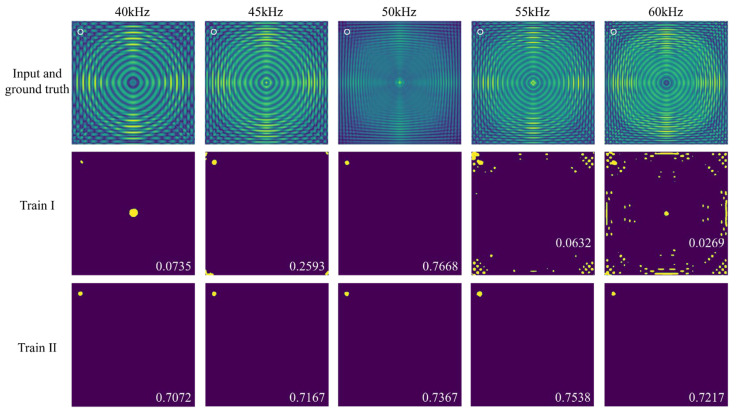
Delamination imaging results for various frequency FDS in scenario 7, comparing UNet++ trained with 50 kHz FDS (Train I) and UNet++ trained with multi-frequency FDS (Train II). The first row displays the model input and ground truth, the second row shows the delamination imaging results of Train I, and the third row presents the delamination imaging results of Train II.

**Figure 14:**
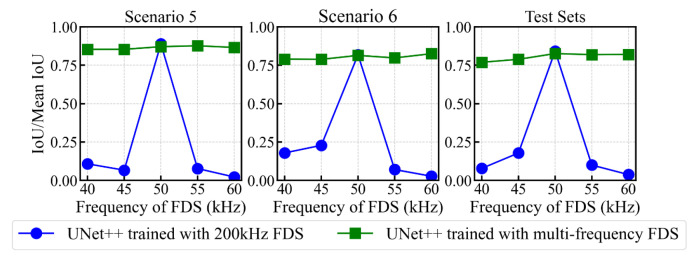
Variation in the IoU of delamination imaging results for various frequency FDS in scenario 5 and scenario 6, along with the mean IoU for various entire test sets composed of specific frequency FDS, comparing UNet++ trained with 50 kHz FDS (Train I) and UNet++ trained with multi-frequency FDS (Train II).

Figure 13 presents the delamination imaging results for the frequency offset FDS in scenario 7, comparing the models from Train I and Train II. The results show that the model trained exclusively with 50 kHz FDS (Train I) performed poorly on frequency offset FDS. Although it could detect delamination, significant artifacts were observed in the images. In contrast, the model trained with multi-frequency FDS (Train II) exhibited a substantial improvement in accuracy when processing frequency offset FDS. This improvement is further confirmed by the results in Figure 14, which show the IoU for delamination imaging across various frequency FDS in scenario 5, scenario 6, and the mean IoU for different test sets composed of specific frequency FDS. While the models in Train I demonstrated a slight advantage in the 50 kHz scenario due to their greater exposure to 50 kHz FDS, the models in Train II achieved significantly higher overall accuracy across the various frequency scenarios, as they had learned to adapt to the changes caused by frequency offsets in FDS. It is worth noting that the performance of the models in Train II was relatively worse in the 40 kHz and 45 kHz scenarios. This can be attributed to the inherent limited spatial resolution of low-frequency FDS in capturing energy distribution details.

Next, the anti-noise ability of the models in Train II was tested. White Gaussian noise, white Laplace noise, and pink noise at levels of 20 dB, 30 dB, and 40 dB were added to the full wavefields in the test set. These noisy full wavefields were then converted into FDS and fed into the models in Train II. The mean IoU of predicted delamination images in various noisy test sets across five folds was computed.

**Figure 15:**
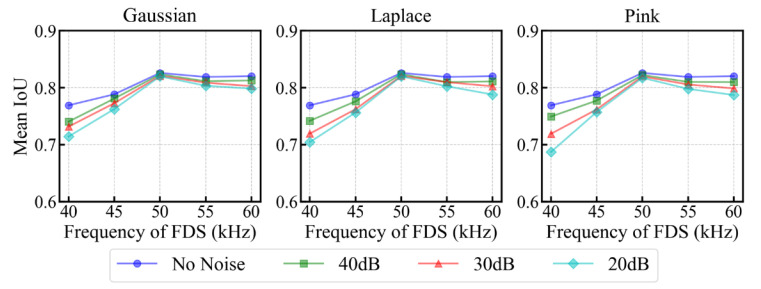
Variation in mean IoU for various test sets composed of specific frequency FDS interfered with 20 dB, 30 dB, and 40 dB white Gaussian noise, white Laplace noise, and pink noise.

Figure 15 illustrates the variation in mean IoU for different test sets composed of specific frequency FDS subjected to 20 dB, 30 dB, and 40 dB white Gaussian noise, white Laplace noise, and pink noise. The impact of noise on the accuracy of delamination imaging is consistent with the trends observed in our self-built dataset (Subsection B of the same section). For the carrier frequency and its surrounding frequencies (45 kHz, 50 kHz, and 55 kHz), the fluctuations in FDS pixel values caused by noise are relatively small, leading to a slight decrease in the mean IoU of delamination imaging. As the frequency offset increases, the energy of the FDS weakens, which makes the pixel disturbances caused by noise more pronounced, resulting in a greater decrease in the mean IoU of delamination imaging.

Table 6 presents the mean IoU of delamination imaging for the test set, which consists of 50 kHz FDS, and compares the models from Train I and Train II. It also includes a comparison with the results reported by Ijjeh et al. [20,21], where the test set consists of RMS data. The results demonstrate that the method proposed in this paper outperforms the others. Although there are differences in the feature types used (FDS vs. RMS), both methods focus on segmenting the energy responses in the wavefield caused by delamination. The superior performance of the model in this paper can be attributed to the image segmentation capabilities of UNet++ and the use of BCED, which helps the model place additional focus on the overlap between the predicted and actual target regions, thus improving the accuracy of delamination imaging. Furthermore, the energy spectrum extraction approach used in this study requires a shorter full wavefield duration than RMS calculation, which helps reduce the cost of dataset construction.

## 5. Conclusions

This paper introduces an end-to-end delamination imaging method for CFRP using 2D FDS and U-Net++. The model was evaluated on both a custom-built simulation dataset and experimental data acquired via SLDV. The results were promising, and the model surpasses the conventional WF technique in detecting the delaminations of different shapes and sizes. In the simulation scenario, the model trained using mixed frequency FDS could accurately predict FDS at different frequencies and showed robustness against three types of synthetic noise: Gaussian, Laplace, and Pink. In the experimental scenario, the model trained solely on simulation data could generalize on the experimental FDS data collected by SLDV and maintained stable performance across FDS frequency variations, with a peak IoU score of 0.7503. Furthermore, we compared the proposed method with those reported by Ijjeh et al. [20,21] on the same public dataset, and our model achieved a higher average IoU score, demonstrating superior delamination imaging performance.

However, in practical applications, collecting large-scale full wavefield data using SLDV can be time-consuming, which limits the real-time detection capabilities of this method. With advancements in laser technology, the data acquisition process is expected to transition from single-point to array-based measurements, significantly reducing the time required for signal collection. Additionally, utilizing deep learning techniques to improve the resolution of sparsely collected full wavefields offers a potential solution to accelerate the full wavefield acquisition process. Recent research has already shown encouraging progress in this research direction [35].

The delamination imaging method proposed in this paper relies on segmenting the changes in spatial energy distribution caused by delamination. While the FDS effectively represents the energy distribution in the full wavefield within the frequency domain, it contains numerous incident and boundary-reflected wave components which can interfere with the model’s segmentation, especially near the edges and corners of the structure. To overcome this issue, future research will focus on extracting feature spectra that eliminate the incident and reflected wave components for model training or exploring the use of more advanced neural network architectures, which could enhance the accuracy and robustness of delamination imaging. In addition, efforts are underway to collect more diverse experimental data with varying delamination types and depths, aiming to further enrich the dataset and optimize the current model.

## Figures and Tables

**Table 1:** Material properties of single-layer CFRP.

ρ (kg/m^3^)	E1 (GPa)	E2 (GPa)	E3 (GPa)	G12 (GPa)	G13 (GPa)	G23 (GPa)	ν12	ν13	ν23
1600	172	11.6	11.6	7.8	7.8	3.9	0.36	0.36	0.55

**Table 2:** Outcome of the dataset splitting.

Composition	Train Set	Validation Set	Test Set	Total
Sample size	1260	360	180	1800

**Table 3:** Imaging result statistics of UNet++ and WF in test set.

Method	IoU			
	Mean	Max	Min	SD
WF	0.4245	0.7400	0.1085	±0.1425
UNet++	0.9449	0.9842	0.7595	±0.0292

**Table 4:** Composition of the remixed training set based on the self-constructed dataset.

Frequency of FDS	140 kHz	160 kHz	180 kHz	200 kHz	220 kHz	240 kHz	260 kHz	Total
Sample size	178	179	184	186	170	178	185	1260

**Table 5:** Composition of the remixed training set derived from the public dataset [22].

Frequency of FDS	40 kHz	45 kHz	50 kHz	55 kHz	60 kHz	Total
Sample size	243	233	250	252	238	1216

**Table 6:** Comparison of imaging statistical results using open dataset.

Method	Mean IoU
ERMSF [20,21]	0.3730
FCN-DenseNet [20,21]	0.6800
Res-UNet [20,21]	0.6640
VGG16 [20,21]	0.5720
PSPNet [20,21]	0.5490
GCN [20,21]	0.7630
UNet++ (this work)	0.8422 (Train I)
	0.8259 (Train II)

## Data Availability

The data supporting the findings of this study are available from the corresponding author upon reasonable request.
